# Co-learner presence and praise alters the effects of learner-generated explanation on learning from video lectures

**DOI:** 10.1186/s41239-022-00363-5

**Published:** 2022-12-08

**Authors:** Zhongling Pi, Caixia Liu, Qian Meng, Jiumin Yang

**Affiliations:** 1 Key Laboratory of Modern Teaching Technology (Ministry of Education), Shaanxi Normal University, No. 199 South Chang’an Road, Yanta District, Xi’an, 710062 Shaanxi Province China; 2 Faculty of Artificial Intelligence in Education, Central China Normal University, No. 152 Luoyu Road, Hongshan District, Wuhan, 430079 Hubei Province China; 3 Jinan Yellow River Bilingual Experimental School, No. 19 Lanxiang Middle Road, Tianqiao District, Jinan, 250031 Shandong Province China

**Keywords:** Video lectures, Explanations, Co-learner presence, Praise

## Abstract

Learning from video lectures is becoming a prevalent learning activity in formal and informal settings. However, relatively little research has been carried out on the interactions of learning strategies and social environment in learning from video lectures. The present study addresses this gap by examining whether learner-generated explanations and co-learner presence with or without nonverbal praise independently and interactively affected learning from a self-paced video lecture about infectious diseases. University students were randomized into viewing either the video with instructor-generated explanations or the same video but generating explanations themselves. Outcomes were assessed by the quality of explanations, learning performance, mental effort, attention allocation, and behavioral patterns. Between-group comparisons showed that, in the absence of a peer co-learner, learning performance was similar in both the instructor-generated and learner-generated explanation groups. However, in the presence of a peer, learner-generated explanation facilitated learning performance. Furthermore, learner-generated explanation in the presence of a co-learner also reduced learners’ mental effort and primed more behaviors related to self-regulation and monitoring. The results lead to the following strong recommendation for educational practice when using video lectures: if students learn by generating their own explanations in the presence of a co-learner, they will show better learning performance even though the learning is not necessarily more demanding, and will engage in more behaviors related to explanation adjustment and self-regulation.

## Introduction

Suppose you are going to watch video lectures on how to prevent COVID-19. Is it better to view the lectures passively, or to view them and generate your own explanations of what you are learning? Research on video lectures suggests that learners show enhanced attention and better learning performance when watching a video lecture using the learning strategy of generating explanations (Fiorella et al., 2020; Pi et al., 2021).

Learning from video lectures has become a prevalent learning activity in both formal and informal educational settings. However, when passively viewing video lectures, many learners struggle to actively make sense of the learning material (i.e., selecting incoming information, organizing it, and integrating it with prior knowledge). One approach that helps learners to actively make sense of the learning material and optimize their learning from video lectures is to generate explanations as they learn (Fiorella & Mayer, 2016). Generating explanations is an active learning strategy that involves constructing statements that clarify the meaning of new knowledge by relating it to prior knowledge, promoting a deeper understanding of the learning material (Pi et al., 2021).

Given the rapid growth of online learning, it is becoming more and more common for learners to engage in online activity simultaneously with their fellow learners (Pi et al., 2020). For example, learners often simultaneously watch the same video lecture via massive open online courses (MOOCs). Studies on peer presence suggest that the mere presence of a peer or simply being aware of the co-learner’s presence can facilitate or inhibit learning performance (Belletier et al., 2019; Skuballa et al., 2019). However, relatively little research has been carried out on the social environment in which learning from video lectures takes place, and even less on the interaction of social environment and learning strategies on learning from video lectures.

### The effects of learner-generated explanation on learning from video lectures

Generative learning theory emphasizes that learner-generated explanations facilitate learning (Chi, 2000). According to the theory, unlike other generative learning strategies (e.g., summarizing, retrieval activities), learner-generated explanation has the goal of making new material personally relevant (Chi, 2000; Fiorella & Mayer, 2016). Therefore, learners are able to fill in missing information, monitor their understanding, and regulate fusions of new information with prior knowledge when discrepancies or deficiencies are detected (Chi, 2000).

Learner-generated explanation has been studied for three decades now. One of the first studies in this area was conducted by Chi et al. (1989), who found that learners spontaneously generated explanations for themselves while reading a book. More recent research documented a positive relation between learner-generated explanation and learning outcomes (Lachner et al., 2021; Lawson & Mayer, 2021). However, much of the research on the effects of learner-generated explanation has focused on traditional classrooms, text, and animation as learning contexts (Lawson & Mayer, 2021; Roelle & Renkl, 2019), and there is a scarcity of research on the effects of learner-generated explanation in the context of video lectures.

Researchers have only recently begun to identify learning strategies that are more effective than passive viewing when learning from video lectures. To our knowledge, only two studies have examined the effects of learner-generated explanation on learning from video lectures (Fiorella et al., 2020; Pi et al., 2021). Both studies confirmed that learner-generated explanation when learning from video lectures enhanced learning performance. Furthermore, Pi et al. (2021) found that, compared with learners watching instructor-generated explanations, those who engaged in learner-generated explanations showed greater neural oscillations related to working memory and attention (increased theta and alpha band power).

### The effects of co-learner presence on learning from video lectures

Research on learning from video lectures with co-learner presence, with both learners engaged in the same online learning activity, has been done in infant (Lytle et al., 2018), school-age (Tricoche et al., 2021), and adult samples (Pi et al., 2020). Evidence in social psychology shows that having a co-learner present who is engaged in the same learning task has considerable positive effects on learning outcomes across a range of learning contexts, including traditional classrooms (Tricoche et al., 2021) and online learning (Skuballa et al., 2019).

The social environment of co-learner presence also has positive effects on learning from video lectures (Huang et al., 2017; Lytle et al., 2018). For example, Li et al. (2014) reported that learners with co-learners who attended the weekly video lectures in one of two MOOCs resulted in higher satisfaction ratings, and both students showed better learning from the video lectures than those without co-learners. In another study, Lytle et al. (2018) reported that learners learned more from video lectures that taught foreign-language sounds when there was a co-learner presence. Compared to learners who learned alone, those who learned with a co-learner exhibited more speech-like vocalizations and were better able to discriminate between the foreign-language sounds.

For more than a century, social psychologists have tried to understand how co-learner presence can help or hinder learning (Zajonc, 1965). According to social presence theory, co-learner presence helps us learn because it primes the learner to adopt the perspectives of others, represent others’ minds, and infer whether and how others understand the incoming information (Jouravlev et al., 2018; Lachner et al., 2021). The learner adjusts explanations and self-regulates based on these mentalizing processes. As a consequence, these self-regulations might enhance learning performance. By contrast, the distraction-conflict theory postulates that co-learner presence triggers a conflict between attention to the ongoing task and attention to the co-learner (Belletier et al., 2019; Skuballa et al., 2019). This attention conflict might lead to cognitive overload, resulting in more mental effort and less learning.

### The effects of praise on learning

Praise is a form of positive feedback. It commends the worth of a person, or expresses approval or admiration after the person shows a desired behavior or meets a goal (Al-Ghamdi, 2017). There is a growing body of literature on feedback that recognizes the importance of praise in learning (Al-Ghamdi, 2017; Zhao & Huang, 2020). The literature has shown that an image of an instructor or even a social robot expressing praise can improve learning performance, for example in math and reading comprehension (Davison et al., 2021). Researchers attribute the mechanisms of these benefits to learners’ increased motivation and positive attitude about new learning and further learning tasks (Maclellan, 2005; Takeue et al., 2013).

Drawing on feedback literature, one may speculate that the presence of a co-learner’s image expressing praise would be a beneficial social environment for learning from video lectures. However, the majority of studies on the effects of praise on learning have been conducted in other learning contexts, such as traditional classrooms (Al-Ghamdi, 2017) or a text-picture presentation on a screen (Zhao & Huang, 2020). It is unclear whether praise affects learning from video lectures, in which the brief presentation of information potentially could create more cognitive load.

### The current study

Past research has largely examined the separate effects of learning strategies (Fiorella et al., 2020; Pi et al., 2021) and the social environment (Lytle et al., 2018) rather than their joint effects on learning from video lectures. Given the rapid growth of video lectures in formal and informal learning, it is important to establish effective learning strategies and understand ideal social environments in which these strategies can be used to foster deep learning. This information will also be critical in order to identify evidence-based ways to optimize performance in this learning context. The present study contributes to this goal by examining whether different learning strategies and social environments independently and interactively affected learning from a video lecture about infectious diseases.

Regarding learning strategies, we compared learner-generated explanation, in which the learner typed out an explanation after each subtopic was presented in a video lecture, to instructor-generated explanation presented as part of the video. Previous studies have indicated that learner-generated explanation while learning from video lectures was more effective than passive viewing (Fiorella et al., 2020; Pi et al., 2021), and in the current study learners passively viewed the instructor-generated explanation presented on a slide in the video. Regarding the social environment, we focused on three social contexts: the presence of a co-learner who gives praise for the learner’s explanation, the presence of a co-learner who does not give praise, and the absence of a co-learner. Co-learner presence in combination with the image of a teacher (Takeue et al., 2013) or robot (Davison et al., 2021) giving praise has been shown to facilitate learning. Finally, it should be noted that, in past research, video lectures were system-paced rather than self-paced. Because system-based pacing is uncommon in the use of video lectures in educational contexts, all video lectures in the current study were self-paced.

The mechanisms underlying the effects of these learning strategies and social environments remain unclear because past research has focused mainly on learning outcomes rather than learning processes. Therefore, in addition to learning performance, we also tested other outcome measures that might provide information about the learning process. These process-related outcomes were: the quality of explanations; self-reported mental effort; attention allocation on the video lecture, the co-learner, and the learner-generated explanation area based on eye tracking measures; and behavioral patterns in initiating different functions in the video area and the explanation area, assessed by log data. These additional outcome variables might provide clues as to why certain combinations of learning strategy and social environment promote learning more than others.

There were five conditions: (1) learner-generated explanation + co-learner + praise; (2) learner-generated explanation + co-learner; (3) learner-generated explanation + no co-learner; (4) instructor-generated explanation + co-learner; and (5) instructor-generated explanation + no co-learner. By comparing the first two conditions, we tested whether praise influenced learning from video lectures. We compared the second and fourth conditions to assess whether learner-generated explanation was more beneficial than passively viewing an instructor-generated explanation for learning in the presence of a co-learner. By comparing the third and fifth conditions, we examined whether learner-generated explanation was more beneficial for learning than passively viewing the instructor-generated explanation when learning from video lectures in the absence of a co-learner.

We utilized generative learning theory, the social presence hypothesis, the distraction-conflict theory, and previous research to make predictions about the effectiveness of learner-generated explanation, co-learner presence, and praise (Chi, 2000; Davison et al., 2021; Fiorella et al., 2020; Lytle et al., 2018; Roscoe & Chi, 2008). Our general prediction was that learning strategy (learner-generated or instructor-generated explanation) would interact with social context (co-learner, co-learner who gives praise, or no co-learner) to predict each outcome. The following hypotheses were proposed:

#### Hypothesis 1

The quality of explanations will be the highest in the presence of a co-learner who gives praise, followed by the presence of a co-learner who does not give praise, then finally by the absence of a co-learner.

#### Hypothesis 2

Learner-generated explanation will benefit learning performance; the benefit will be highest in the presence of a co-learner who gives praise, followed by the presence of a co-learner who does not give praise. Instructor-generated explanation will be less beneficial for learning performance, with lower scores in the presence of a co-learner, and the lowest scores in the absence of a co-learner.

#### Hypothesis 3

Learner-generated explanation will increase self-reported mental effort; the scores will be highest in the presence of a co-learner who gives praise, followed by the presence of a co-learner who does not give praise. Instructor-generated explanation will produce lower mental effort, with lower scores in the presence of a co-learner, and the lowest scores in the absence of a co-learner.

#### Hypothesis 4

Learner-generated explanation will enhance attention to the video lectures as assessed by eye movement data; the benefit will be highest in the presence of a co-learner who gives praise, followed by the presence of a co-learner who does not give praise. Instructor-generated explanation will be less beneficial, with lower attention in the presence of a co-learner, and the lowest attention in the absence of a co-learner.

#### Hypothesis 5

Learner-generated explanation will enhance attention to the co-learner area of the screen, as measured by eye movement data; the greatest enhancement will be in the presence of a co-learner who gives praise, followed by being in the presence of a co-learner who does not give praise, and then the instructor-generated explanation in the presence of a co-learner.

#### Hypothesis 6

The presence of a co-learner will enhance attention to the learner-generated explanation area of the screen, as measured by eye movement data; the greatest enhancement will be in the condition of the learner-generated explanation with the presence of a co-learner who gives praise, followed by the learner-generated explanation with the presence of a co-learner who does not give praise, and then the learner-generated explanation in the absence of a co-learner.

#### Hypothesis 7

Learner-generated explanation will enhance behaviors related to explanation adjustments and self-regulation, as measured by log data in the video area and the learner-generated explanations area; the highest number of behaviors related to explanation adjustments and self-regulation will be in the presence of a co-learner who gives praise, followed by in the presence of a co-learner who does not give praise, then in the absence of a co-learner. Instructor-generated explanation will be less helpful in enhancing learning in the presence of a co-learner, with fewer behaviors related to explanation adjustments and self-regulation, and the fewest behaviors related to explanation adjustments and self-regulation will be observed in the absence of a co-learner.

## Methods

### Participants and design

A priori power analysis was conducted to determine the appropriate sample size. The effect sizes of an *F*-test were 0.10 (small), 0.25 (medium), and 0.40 (large; Cohen, 1988). G*Power (ANCOVA: *f* = 0.4, α = 0.05, power = 0.90, numerator *df* = 4, number of groups = 5) indicated that 102 participants would be sufficient for the planned analyses. A total of 141 undergraduate (*n* = 69) and postgraduate (*n* = 72) students from a Chinese university were recruited to participate in the experiment, and their age ranged from 17 to 27 years old (*M*_age_ = 21.45, *SD*_age_ = 2.02; 122 females). They majored in a wide variety of fields, including psychology, educational technology, philosophy, history, and geography. Most of them had more than one year’s experience in using video conferencing software (e.g., Tencent Video Conferencing); all participants had a fair amount of online learning experience before taking part in this experiment. All participants had normal or corrected-to-normal vision. Participants gave their informed consent before starting the experiment, and received 20 RMB after completing the experiment. The study protocol was approved by the local ethics committee. The experiment used a between-subjects design in which participants were randomly assigned to one of five conditions: (1) learner-generated explanation + co-learner + praise (LE + C-L + Pr, *n* = 27), (2) learner-generated explanation + co-learner (LE + C-L, *n* = 29), (3) learner-generated explanation + no co-learner (LE + NC-L, *n* = 28), (4) instructor-generated explanation + co-learner (IE + C-L, *n* = 29), and (5) instructor-generated explanation + no co-learner (IE + NC-L, *n* = 28).

### Materials

**Fig. 1:**
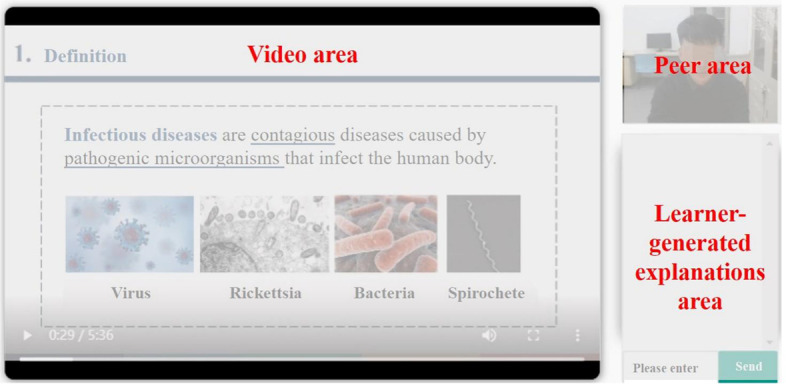
The three areas of the screen (translated from Chinese)

All materials were programmed in HTML with embedded JavaScript in a computer-based learning environment. The screen was divided into three boxes: video box, co-learner box, and explanation box (Fig. 1). Each of the three boxes corresponded to a defined area of interest (AOI) in the eye tracking measures. The instructor’s image did not appear on the screen. Slides were shown on the screen while a local professor taught five subtopics related to infectious diseases: the definition of infectious disease, basic characteristics of infectious disease, pathogenesis, epidemic process, and prevention. In the learner-generated explanation (LE) conditions, participants were prompted after each subtopic to write an explanation. In the instructor-generated explanation (IE) conditions, the instructor provided an explanation after teaching each subtopic. For example, “Infectious diseases are contagious diseases caused by pathogenic microorganisms (virus, rickettsia, bacteria, spirochetes, etc.) that infect human body.” The video lasted 5:36 min, and participants could pause or restart the video at any time.

**Fig. 2:**
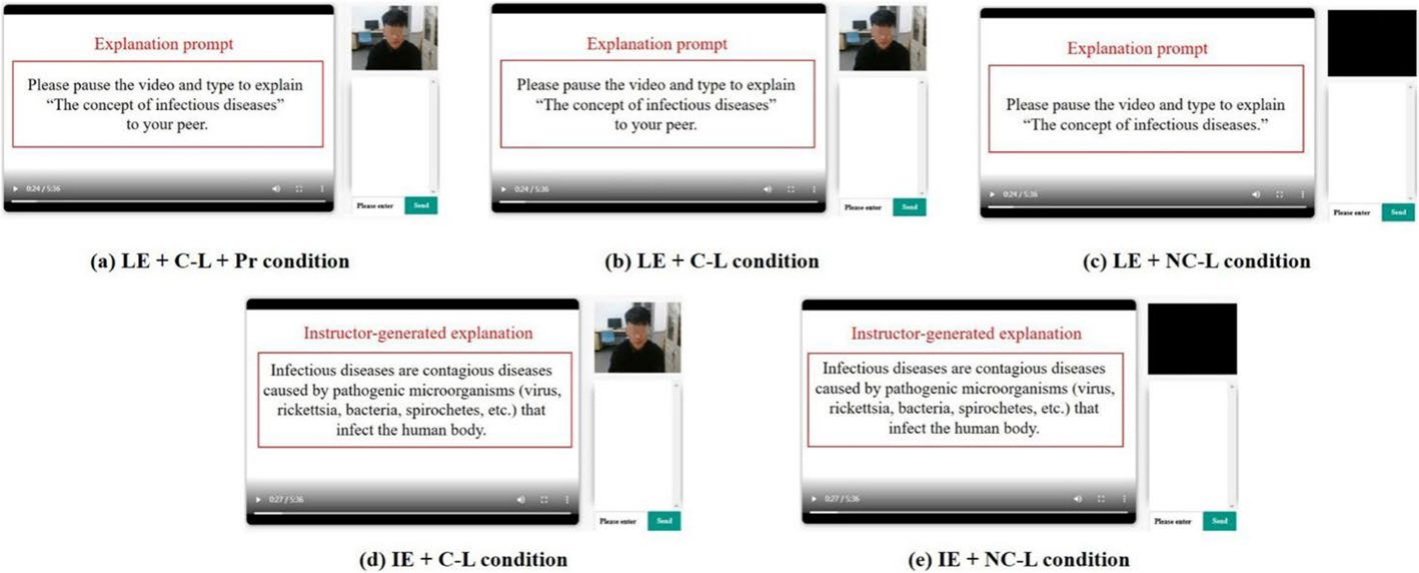
Screenshots of the five conditions (translated from Chinese). The red words and lines did not appear in the video lectures

In the LE + C-L + Pr condition, an explanation prompt was presented after each subtopic, and the participant was asked to type an explanation of the subtopic for their co-learner. A live video showed a real male co-learner (i.e., an experimenter) of a similar age as the participants. They were presented in the co-learner area and gave the participant non-verbal praise (nodding their head or smiling) to express approval or admiration of the learner-generated explanation. See Fig. 2a. The LE + C-L condition was similar to the LE + C-L + Pr condition, the only difference being that the co-learner in the live video did not give the participant non-verbal praise. See Fig. 2b. In the LE + NC-L condition, an explanation prompt was presented after each subtopic, and the participant was asked to type their explanation in the learner-generated explanation area. The co-learner area was black. See Fig. 2c. In the IE + C-L condition, an instructor-generated explanation was presented after each subtopic, and in the co-learner area a live video presented a real male co-learner (i.e., an experimenter) of a similar age as the participants. See Fig. 2d. In the IE + NC-L condition, an instructor-generated explanation was presented after each subtopic and the co-learner area appeared as an empty black square. See Fig. 2e.

### Instruments and measures

#### Prior knowledge test

A prior knowledge test developed by the professor in the video was used to assess participants’ general knowledge of “infectious disease” at the start of the study. The prior knowledge score was used as a control variable. The test was made up of one open-answer question: “What measures can be taken to prevent infectious diseases?” (6 points). Answers were assessed by two trained raters, with high inter-rater reliability (*r* = 0.91, *p* < 0.001). The result of one-way ANOVA showed that there was no significant difference in the prior knowledge test scores across the five conditions, *F*(4, 136) = 0.29, *p* = 0.882, η^2^ = 0.01.

#### Explanation coding scheme

An explanation coding scheme was used to assess the quality of explanations generated by the LE + C-L + Pr, LE + C-L, and LE + NC-L groups, which included two categories: incorrect items and covered main content items (Hiller et al., 2020). Two independent coders counted the explanations based on 20% of the data, and the inter-coder reliability was high (*r*_incorrect_ = 0.90, *r*_covered main content_ = 0.88, *p*s < 0.001). Then, one coder coded all the remaining data.

#### Learning performance test

A learning performance test was used to assess the participants’ mastery of the knowledge presented in the video. It included one multiple-choice item (four options; 2 points for choosing only the correct option), and four fill-in-the-blank items (1 point for each blank, total of 10 points). The total possible score was 12 points. The test had high internal consistency (Cronbach’s α = 0.61).

#### Mental effort

Mental effort was measured by one item that the participants rated on a Likert scale ranging from 1 (an extremely small amount) to 9 (an extremely large amount): “How much mental effort have you spent on learning just now?” (Paas & Van Merriënboer, 1994).

#### Log data coding system

A coding system was developed using the participants’ log data to capture behaviors related to explanation adjustments and self-regulation in initiating different functions in the video area and the learner-generated explanations area (see Table 1; Li, 2019). Two experts who were proficient in video learning behavior analysis verified the feasibility of the coding system. The two trained coders showed high inter-rater reliability in coding all behaviors (*Kappa* > 0.99 based on 20% of the data).

**Table 1:** The log data coding system

Code	Behavior	Description
Pl	Play	Clicked the video section to play the video
Pa	Pause	Clicked the video section to pause the video
GF	Go forward	Clicked the progress bar to jump to a later time point
GB	Go backward	Clicked the progress bar to jump to a previous time point
TE	Type explanation	Clicked the enter box to type in the explanation
SE	Send explanation	Clicked the send button to send the explanation

### Apparatus and eye movement data analysis

A Tobii T120 eye tracker was used to record participants’ eye movements with a sampling rate of 120 Hz. The web page was presented on a 17-inch monitor with a resolution of 1280 × 1024 and a refresh rate of 75 Hz. The participants sat 60 cm away from the monitor.

To measure participants’ attention allocation, three areas of interest (AOIs) were created on the web page: the video lecture area, the co-learner area, and the learner-generated explanation area, each corresponding to one of the three visible boxes on the screen (see Fig. 1). We used eye movement data to calculate the percentage fixation duration on each AOI (i.e., the fixation duration on each area divided by the total fixation time).

### Lag sequential analysis

To understand the learning process in the different experimental conditions, we recorded learning behaviors (log data on different parts of the screen) during the video lectures and used lag sequential analysis (LSA) with GSEQ 5.1 software to identify behavioral patterns (Bakeman & Gottman, 1997). LSA was used to test the statistical significance of differences in behavioral patterns across the five conditions. First, the entire sequence of coded behaviors in each condition was imported in GSEQ 5.1 and saved as an independent document. Then, GSEQ 5.1 was used to identify smaller sequences (patterns) based on the frequency and adjustment residual of each behavior, with a *Z*-score > 1.96 indicating that the sequence is statistically significant (Bakeman & Gottman, 1997). Finally, we plotted five behavior transition diagrams based on all sequences that reached statistical significance.

### Procedure

The experiment was conducted in an eye movement lab and lasted about 40 min. At the beginning of the experiment, the participants were introduced to the experimental procedure and they signed the informed consent form (5 min). They then completed the demographic questionnaire (e.g., gender, age, and major) and prior knowledge test (5 min). At this point, the participants were randomly assigned to one of the five conditions (i.e., LE + C-L + Pr, LE + C-L, LE + NC-L, IE + C-L, and IE + NC-L). As they took part in the video lecture part of the experiment, their eye movements and log data were recorded. Afterward, they completed the mental effort scale and learning performance test (10 min).

## Results

The scores on the measures of learning performance and mental effort across the five conditions all showed a normal distribution (skewness < 1.00, kurtosis < 1.00; *p*s > 0.05) and homogeneity of variance (*p*s > 0.05). One-way analyses of covariance (ANCOVAs) were conducted with these two variables as dependent measures and the prior knowledge test score as the covariate. However, the quality of explanations and the percentage fixation duration on the three AOIs did not show a normal distribution (*p*s < 0.001), and thus non-parametric tests were conducted on these dependent variables. The descriptive statistics for all dependent variables are shown in Table 2.

**Table 2:** Means (*M*) and standard deviations (*SD*) for all dependent variables

Dependent variables	LE + C-L + Pr (*n* = 27)		LE + C-L (*n* = 29)		LE + NC-L (*n* = 28)		IE + C-L (*n* = 29)		IE + NC-L (*n* = 28)	
	*M*	*SD*	*M*	*SD*	*M*	*SD*	*M*	*SD*	*M*	*SD*
Prior knowledge	2.30	1.42	2.31	1.38	2.63	1.17	2.33	1.45	2.32	1.39
Quality of explanations										
Incorrect	0.93	0.96	0.86	0.95	0.86	1.11	–	–	–	–
Covered main content	4.41	0.93	4.17	0.93	4.14	1.11	–	–	–	–
Learning performance	7.09	2.28	6.52	2.85	6.89	2.23	5.29	2.23	5.82	2.07
Mental effort	7.07	1.17	6.21	1.40	7.04	1.14	6.55	1.33	7.11	1.23
Percentage fixation duration (%)										
Video lecture area	85.61	6.16	87.27	6.75	88.18	6.21	98.42	1.98	99.59	0.50
Co-learner area	2.57	1.47	1.51	1.30	–	–	1.24	1.91	–	–
Learner-generated explanations area	11.81	5.46	11.22	6.59	11.76	6.21	–	–	–	–

### Quality of explanations

Hypothesis 1 was that the quality of explanations would be the highest in the presence of a co-learner who gives praise. Two Kruskal–Wallis *H* tests were conducted on the quality of incorrect and covered main content explanations separately. However, the results showed no significant difference in the quality of incorrect explanations (*H* = 0.40, *p* = 0.820) and covered main content explanations (*H* = 1.31, *p* = 0.519), which was inconsistent with our hypothesis.

### Learning performance

**Fig. 3:**
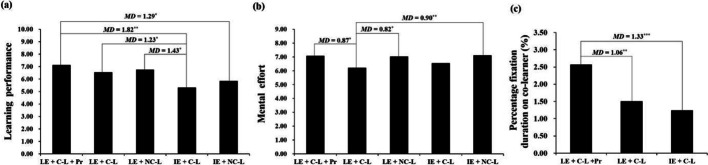
Differences in (**a**) learning performance, (**b**) mental effort, and (**c**) percentage fixation duration on the co-learner area across the groups (*p < 0.05; **p < 0.01; ***p < 0.001)

Hypothesis 2 was that the condition of learner-generated explanation in the presence of a co-learner who gave praise would have the greatest benefits on learning performance. The ANCOVA results showed a significant difference across the five groups, *F*(4, 135) = 3.03, *p* = 0.020, η_p_^2^ = 0.08. Further *LSD* post hoc tests (Fig. 3a) showed that, as expected, the LE + C-L + Pr group showed the highest learning performance. Also as expected, the scores in this group were significantly higher than the scores in both of the instructor-generated explanation groups (for the IE + NC-L group: *MD* = 1.29, *p* = 0.034; for the IE + C-L group: *MD* = 1.82, *p* = 0.003). Moreover, the LE + C-L group (*MD* = 1.23, *p* = 0.036) and the LE + NC-L group (*MD* = 1.43, *p* = 0.017) had significantly better learning performance than the IE + C-L group. However, contrary to our hypothesis, there was not a significant difference between the LE + C-L group and the LE + NC-L group (*MD* = 0.19, *p* = 0.744). In general, the three learner-generated explanation groups showed better learning performance than the two instructor-generated explanation groups. Together, these results partially supported our hypothesis.

### Mental effort

Hypothesis 3 was that the condition of the learner-generated explanation in the presence of a co-learner who gave praise would produce the highest mental effort scores. The ANCOVA results showed a significant difference in mental effort scores across the five groups, *F*(4, 135) = 2.84, *p* = 0.027, η_p_^2^ = 0.08. As expected, the scores in the LE + C-L + Pr group were significantly higher than those in the LE + C-L group (*MD* = 0.87, *p* = 0.011; Fig. 3b), suggesting that when the participant was generating explanations, more mental effort was used when the co-learner gave praise than when they did not*.* However, the two groups with no co-learner also reported higher mental effort than the LE + C-L group (for LE + NC-L: *MD* = 0.82, *p* = 0.015; for IE + N-CL: *MD* = 0.90, *p* = 0.008). The latter results are more difficult to interpret. In general, it appeared that having no co-learner produced higher mental effort than when a co-learner was present; when a co-learner was present, more mental effort was used when the co-learner gave vs. did not give praise.

### Attention allocation

To analyze group differences in students’ attention allocation, Kruskal–Wallis *H* tests were conducted on the percentage of fixation duration on each of the three AOIs (video lecture, co-learner, and learner-generated explanation areas).

#### Percentage fixation duration on video lecture area

Hypothesis 4 was that participants providing learner-generated explanations in the presence of a co-learner who gave praise would pay the greatest attention to the video area. The Kruskal–Wallis *H* test on the percentage of fixation duration on the video area showed a significant difference across the five groups (*H* = 99.23, *p* < 0.001). However, contrary to our hypothesis, further post hoc tests showed that the instructor-generated explanation groups (IE + C-L and IE + NC-L) showed a higher percentage of fixation duration on the video lecture area than the learner-generated explanation groups (LE + C-L + Pr, LE + C-L, and LE + NC-L; *p*s < 0.001). Furthermore, there was no significant difference between the two instructor-generated explanation groups, nor among the three learner-generated explanation groups (*p*s > 0.05). In general, it seemed that the two instructor-generated explanation groups paid greater attention to the video than the three learner-generated explanation groups, which did not support our hypothesis.

#### Percentage fixation duration on co-learner area

Hypothesis 5 was that participants who provided learner-generated explanations would pay greater attention to the co-learner area when the co-learner gave praise than when they did not give praise. As the co-learner was not present in either the IE + NC-L or LE + NC-L groups, a Kruskal–Wallis *H* test was performed on the percentage fixation duration on the co-learner area among the other three groups (LE + C-L + Pr, LE + C-L, and IE + C-L). The result was significant, *H* = 17.48, *p* < 0.001. As expected, further post hoc tests (Fig. 3c) showed that the LE + C-L + Pr group had a higher percentage of fixation duration on the co-learner area than the LE + C-L group (*MD* = 1.06, *p* = 0.007) and the IE + C-L group (*MD* = 1.33, *p* < 0.001). However, contrary to our hypothesis, no significant difference was found between the LE + C-L and IE + C-L groups (*p* = 0.151). In general, presenting a co-learner was not necessary to enhance the attention given to the co-learner area, but presenting a co-learner who gave praise enhanced the attention given to the co-learner area, which supported our hypothesis.

#### Percentage fixation duration on learner-generated explanation area

Hypothesis 6 was that, among participants who provided learner-generated explanations, those in the presence of a co-learner who gave praise would show the greatest attention to the learner-generated explanation area. As the instructor-generated explanation groups were not asked to type an explanation, a Kruskal–Wallis *H* test was conducted on the percentage of fixation duration on the learner-generated explanation area only among the learner-generated explanation groups (LE + C-L + Pr, LE + C-L, and LE + NC-L). However, the results showed no significant differences among the various groups (*H* = 0.92, *p* = 0.632). In general, the presence of the co-learner did not enhance attention given to the learner-generated explanation area of the screen, contrary to our hypothesis.

### Behavioral patterns

Hypothesis 7 was that participants who provided learner-generated explanations in the presence of a co-learner who gave praise would show the most behaviors related to explanation adjustments and self-regulation. Lag sequence analyses of the log data were conducted for each of the five groups. Tables 3, 4, 5, 6, 7 show the adjusted residual results of the sequential analysis, with a *Z*-score > 1.96 indicating that the behavior path is significant. Each row represents an initial behavior and each column represents a follow-up behavior.

**Table 3:** Results of sequential analysis of log data in the LE + C-L + Pr group

*Z*-score	Pa	Pl	GF	GB	TE	SE
Pa	− 8.40	− 3.39	− 2.71	0.58	**19.50**	− 7.63
Pl	**22.38**	− 8.02	− 0.64	− 0.01	− 7.36	− 7.44
GF	− 0.25	− 0.57	**5.35**	**2.92**	− 1.23	− 2.41
GB	0.55	− 0.67	**7.57**	0.39	1.11	− 4.61
TE	− 7.22	− 7.21	− 2.71	0.06	− 8.56	**25.54**
SE	− 7.32	**20.79**	− 1.84	− 2.25	− 4.33	− 6.68

Pa = Pause; Pl = Play; GF = Go forward; GB = Go backward; TE = Type explanation; SE = Send explanation. Values in bold indicate that the behavior path is significant (*p* < 0.05)

**Table 4:** Results of sequential analysis of log data in the LE + C-L group

*Z*-score	Pa	Pl	GF	GB	TE	SE
Pa	− 7.48	− 3.37	− 3.86	**3.17**	**12.81**	− 2.16
Pl	**18.00**	− 7.50	− 0.25	0.04	− 3.37	− 7.24
GF	− 1.23	− 1.99	**13.20**	0.74	− 2.54	− 4.47
GB	0.45	− 2.59	0.69	**3.99**	**3.10**	− 5.08
TE	− 3.71	− 5.82	− 4.52	− 2.10	− 7.59	**22.93**
SE	− 6.77	**21.36**	− 1.40	− 5.23	− 2.67	− 6.41

Pa = Pause; Pl = Play; GF = Go forward; GB = Go backward; TE = Type explanation; SE = Send explanation. Values in bold indicate that the behavior path is significant (*p* < 0.05)

**Table 5:** Results of sequential analysis of log data in the LE + NC-L group

*Z*-score	Pa	Pl	GF	GB	TE	SE
Pa	− 8.80	− 1.57	− 2.88	0.98	**18.79**	− 7.25
Pl	**22.18**	− 8.86	− 0.85	− 0.18	− 6.80	− 7.13
GF	− 1.06	− 1.73	**8.58**	1.87	− 0.49	− 3.21
GB	− 0.27	0.05	**4.23**	**2.58**	− 0.22	− 4.27
TE	− 7.30	− 7.45	− 3.17	0.01	− 7.12	**26.01**
SE	− 6.69	**20.91**	− 1.19	− 4.22	− 5.48	− 5.87

Pa = Pause; Pl = Play; GF = Go forward; GB = Go backward; TE = Type explanation; SE = Send explanation. Values in bold indicate that the behavior path is significant (*p* < 0.05)

**Table 6:** Results of sequential analysis of log data in the IE + C-L group

*Z*-score	Pa	Pl	GF	GB
Pa	− 5.45	**16.89**	− 6.91	− 3.11
Pl	**11.77**	− 5.52	− 6.47	1.30
GF	− 4.52	− 6.99	**11.07**	− 1.83
GB	− 1.60	− 3.36	0.75	**4.33**

Pa = Pause; Pl = Play; GF = Go forward; GB = Go backward. Values in bold indicate that the behavior path is significant (*p* < 0.05)

**Table 7:** Results of sequential analysis of log data in the IE + NC-L group

*Z*-score	Pa	Pl	GF	GB
Pa	− 4.39	**12.64**	− 4.96	− 3.29
Pl	**8.09**	− 5.78	− 3.61	**2.02**
GF	− 1.89	− 4.99	**9.54**	− 3.78
GB	− 2.56	− 0.66	− 1.81	**5.50**

Pa = Pause; Pl = Play; GF = Go forward; GB = Go backward. Values in bold indicate that the behavior path is significant (*p* < 0.05)

**Fig. 4:**
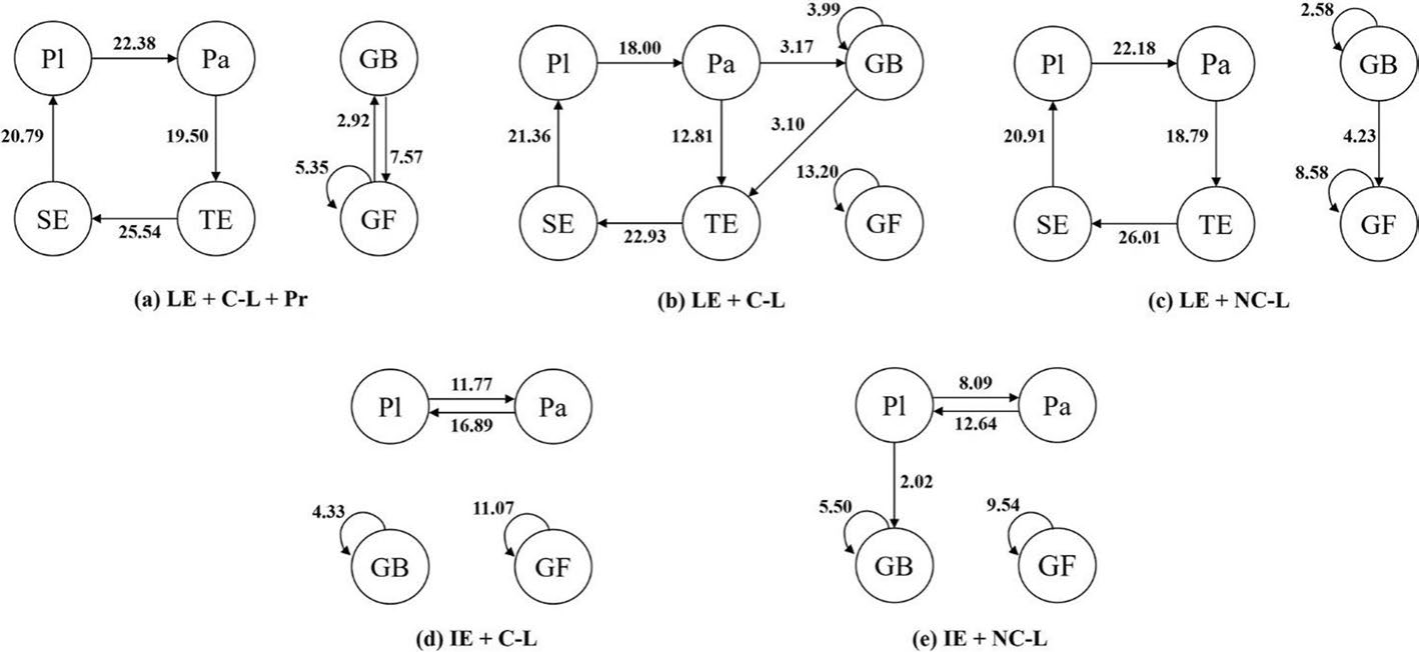
Behavior transition diagrams for the (**a**) LE + C-L + Pr, (**b**) LE + C-L, (**c**) LE + NC-L, (**d**) IE + C-L, and (**e**) IE + NC-L groups. Pl = Play; Pa = Pause; GF = Go forward; GB = Go backward; TE = Type explanation; SE = Send explanation

All the significant behavior sequences were plotted to form a behavioral transition diagram for each of the five groups (see Fig. 4). The numbers represent the *Z*-scores, and the arrows point in the direction of the transfer. The LE + C-L + Pr and LE + NC-L groups had seven significant behavior sequences, the LE + C-L group showed eight significant behavior sequences, the IE + C-L group showed four significant behavior sequences, and the IE + NC-L group had five significant behavior sequences.

**Fig. 5:**
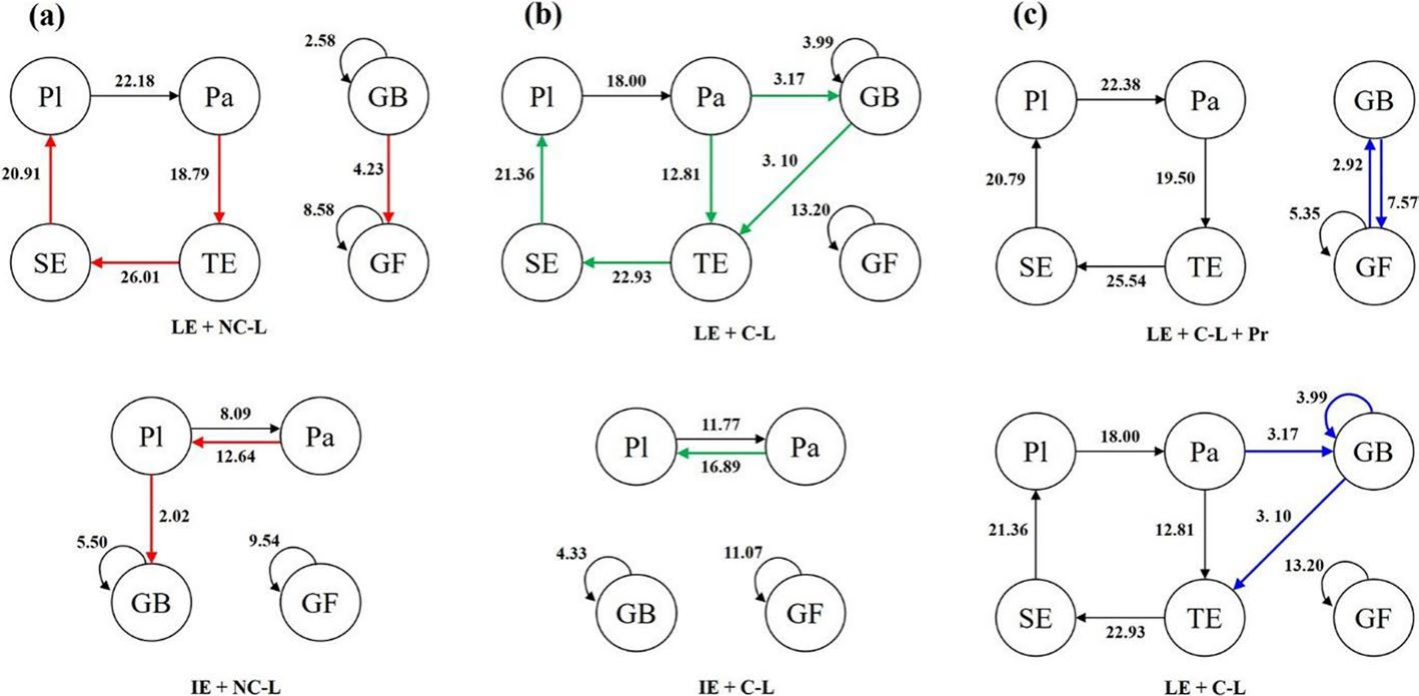
Three comparisons of the behavior sequences: (**a**) LE + NC-L vs. IE + NC-L, (**b**) LE + C-L vs. IE + C-L, and (**c**) LE + C-L +Pr vs. LE + C-L. Pl = Play; Pa = Pause; GF = Go forward; GB = Go backward; TE = Type explanation; SE = Send explanation

We then examined whether certain behavior sequences were more common in one group compared to a relevant comparison group. We made three comparisons to address the following three questions (see Fig. 5):

First, when the co-learner was absent in both groups, did the learner-generated and instructor-generated explanation groups show different patterns of behavior? To test the effects of the learner-generated explanation in the absence of a co-learner, we compared LE + NC-L with IE + NC-L groups (see Fig. 5a). We found that the IE + NC-L group showed more Pa → Pl and Pl → GB behavior sequences, but fewer GB → GF behavior sequences than the LE + NC-L group. That might imply that the learners in the IE + NC-L group tended to process the learned information over again. Because learners in the IE + NC-L group re-watched the video more often than those in the LE + NC-L group, it is possible they were able to engage in deeper learning of the video content. Furthermore, the LE + NC-L group showed significant Pa → TE and SE → Pl behavior sequences, suggesting that these learners tended to pause the video before they typed explanations; then, after they sent the explanations, they continued to play the video.

Second, when a co-learner was present in both groups, did the learner-generated and instructor-generated explanation groups show different patterns of behavior? We compared the behavior sequences in the IE + C-L and LE + C-L groups (see Fig. 5b). While watching the video, the LE + C-L group showed more Pa → GB behavior sequences but fewer Pa → Pl behavior sequences than the IE + C-L group. This might imply that the LE + C-L group tended to process the learned information a second time over. Learners in the LE + C-L group rewound the video more often than those in the IE + C-L group, possibly resulting in deeper processing of the video content. Furthermore, the LE + C-L group showed Pa → TE, GB → TE, and SE → Pl behavior sequences, suggesting that those learners tended to pause and rewind the video before they typed explanations; then, after they sent the explanations, they continued to play the video.

Third, when both groups use the learner-generated explanation in the presence of a co-learner, did adding praise result in different behavior sequences? We compared the behavioral sequences found in the LE + C-L + Pr and LE + C-L groups (see Fig. 5c). The LE + C-L group showed more Pa → GB and GB → GB behavior sequences but fewer GB → GF and GF → GB behavior sequences than the LE + C-L + Pr group. That is, while the LE + C-L group repeatedly re-watched segments, the LE + C-L + Pr group tended to “jump around,” perhaps looking for specific pieces of information but not having the chance to deeply process the information. When typing explanations, the LE + C-L group showed more GB → TE behavior sequences than the LE + C-L + Pr group, suggesting that the LE + C-L group’s explanations were more closely tied to the video content.

## Discussion

This study focused on the interaction effects of learning strategies and social environment on learning from video lectures, assessed by the quality of explanations, learning performance, mental effort, attention allocation, and behavioral patterns. Comparing the IE + NC-L and LE + NC-L groups, we found that in the absence of a co-learner, learning performance was similar in the instructor-generated and learner-generated explanation groups. These results were inconsistent with previous studies showing that learner-generated explanation enhanced learning performance compared to passively viewing a video lecture with an instructor-generated explanation (Fiorella et al., 2020; Pi et al., 2021).

A possible interpretation of this inconsistent result has to do with whether the learners watched a system-paced or self-paced video. Fiorella et al. (2020) and Pi et al. (2021) found that learner-generated explanations had a positive effect on learning from a system-paced video. By contrast, we did not find a similar positive effect on learning from a self-paced video in which learners could self-regulate and adjust the pace of the lectures. The eye movement data and log data may also help to explain our results. Learners who generated explanations spent less time fixated on the video lectures, and showed fewer pause → play → go backward behavior sequences, but more go backward → go forward behavior sequences than learners who passively viewed instructor-generated explanations. These additional pause → play → go backward sequences suggest that learners viewing instructor-generated explanations had multiple opportunities to re-learn the information as they went backward and forward within the video. Previous studies have shown that the pause and go backward behavior sequence is related to self-regulation, explanation adjustment, and metacognition (Liou, 2000; Sweeney, 2018). Therefore, these behaviors when watching a video with instructor-generated explanations may have the same degree of benefit on learning performance as the learner-generated explanation strategy.

Interestingly, by comparing the LE + C-L group with the IE + C-L group, we found that in the presence of a co-learner, learners who generated explanations showed significantly better learning performance than those who passively viewed instructor-generated explanations. These results are in line with generative learning theory and findings of previous studies (Chen et al., 2015; Chi, 2000; Tsegay, 2015). Generative learning theory suggests that learners who generate their own explanations are more engaged in seeking information and monitoring, which leads to better learning performance. Previous studies have shown that China has been moving from instructor-centered education to learner-centered education over the past several decades, and learners are able to learn collaboratively and actively engage in learning (Chen et al., 2015; Tsegay, 2015; Wang, 2011). The results based on log data are consistent with this possibility. Learners who generated their own explanations showed more play → go backward → typing explanations and pause → typing explanations sequences than those who watched the instructor-generated explanations. The results implied that learner-generated explanation enhanced learners’ rewind, resulting in deeper processing of the video content. Previous studies also suggested that learners’ interaction behaviors (e.g., sharing ideas) were greatly affected by their trust in the instructors and perceptions of the instructors towards learners’ participation in learner-centered learning environments (Gregory & Ripski, 2008; Tsegay, 2015). Future work is needed to examine whether learners’ trust in instructors and perceptions of instructors towards learners’ participation moderates the effects of learner-generated explanation and instructor-generated explanation on learning from video lectures.

Further comparing the LE + C-L group with the LE + NC-L group, the log data showed there was a more common behavior chain of pause → go backward → type explanation in the LE + C-L group. These results suggest that co-learner presence increased learners’ behaviors related to generating and adjusting their explanations. Previous studies have shown that when learning alone, learners adopt an egocentric perspective in interpreting and formulating learning materials; when learning in the presence of others (e.g., a co-learner), they adopt the perspectives of anyone present (Jouravlev et al., 2018; Lachner et al., 2021; Rueschemeyer et al., 2015). Therefore, learners infer the co-learner’s mental activities (e.g., whether and how they understand the learning materials). In the current study, learners were informed that their explanations could be seen by the co-learner. These anticipation processes could engage learners in behavioral adjustments for others, for instance leading learners to generate additional elaborations or explanations (Lachner et al., 2021). This might be a reason why learner-generated explanation was an effective learning strategy in the presence of a co-learner, but not in the absence of a co-learner.

Finally, there was no difference in learning performance between the LE + C-L + Pr and LE + C-L groups. The eye movement data showed that learners who received praise fixated on the co-learner more than those who did not receive praise, but this did not translate into improved learning. This result was unexpected given previous results showing that an image displaying praise increased learners’ motivation and positive attitude about new learning and future learning tasks (Maclellan, 2005; Takeue et al., 2013). It is possible that in our study the praise did not increase motivation and positive attitude to the extent that they would affect learning. Further work is needed to test the cognitive, metacognitive, and motivational mechanisms underlying the effects of learner-generated explanation, co-learner presence, and praise on learning from video lectures.

A second possible reason that praise did not contribute to learning in this study has to do with how learners interacted with the self-paced video. LSA results showed that learners in the LE + C-L + Pr group had more go backward → go forward and go forward → go backward behavior sequences, but fewer pause → go backward → type explanation and go backward → go backward sequences. These results suggest that praise increased learners’ quick searches within the video, but not the deep processing of the kind needed for them to generate and adjust their explanations. Furthermore, learners in the LE + C-L group achieved as good learning performance as those in the LE + C-L + Pr group, but they invested a similar amount of mental effort. This underscores the possibility that having a co-learner present without praise facilitates learning in ways that are not necessarily more demanding but are more effective.

Several features of this study limit the conclusions we can draw about how learning strategies and social environment interact to influence learning from video lectures. First, we did not control students’ personalities, which might have influenced their responses to the co-learner presence and their response to peers’ ideas. A meta-analysis found that personality was a substantial moderator of the effect of others’ presence on a range of behaviors, including learning (Uziel, 2007). For example, extroverted individuals tend to have a positive interpretation of others’ presence, whereas neurotic individuals tend to have a negative interpretation (Ku et al., 2020; Pang & Wu, 2021). More recent studies also found that learners’ openness to experiences influenced their attention to peers’ ideas and their quality of generated ideas when generating ideas with peers, which might influence whether they could benefit from the co-learner presence (Pi et al., 2019, 2022). Therefore, further research should examine whether these and other students’ personality traits moderate the effects of others’ presence on learning from video lectures. Second, we did not test the role of learners’ online learning experience and information literacy, which could have influenced learners’ behavioral patterns as they viewed the video lectures. Previous studies have shown that learners’ online learning experience and information literacy influence their learning strategies and performance (Mac Callum et al., 2014; Wang et al., 2013). For example, a study by Wang et al. (2013) found that the number of previous online courses taken affected the learning strategies of online learning (i.e., elaboration, metacognitive self-regulation, and time management). Learners with more experience in taking online courses used more effective learning strategies. Therefore, further research should examine whether online learning experience and information literacy play a moderation role in learning from video lectures. Third, we failed to find a difference in the quality of learner-generated explanations. One possible reason could be that learners are not good at providing explanations without experience and prior knowledge, which can lead them to generate simpler explanations. In the future, learners will be provided with more detailed instructions on how to generate explanations, and a more scientific coding scheme will be adopted. For instance, some studies assessed the quality of generated explanations with consideration of specific aspects such as comprehensiveness and elaborations (Jacob et al., 2020; Lachner et al., 2017).

In conclusion, we found that in the presence of a peer, learner-generated explanation facilitated learning performance. Furthermore, learner-generated explanation in the presence of a peer also reduced learners’ mental effort and primed more behaviors related to self-regulation, explanation adjustment, and monitoring. The findings extend our understanding of learning outcomes and processes associated with learning strategies and the social environment, enabling us to identify more optimal ways to learn from video lectures. Future research should continue to identify the boundary conditions of the effects of learning strategies and the social environment on learning from video lectures. This issue is very relevant in the field of education, where there is growing interest in optimizing learning from video lectures by including a co-learner (Fiorella et al., 2020; Lytle et al., 2018; Pi et al., 2021). The results lead to a strong recommendation for educational practice when using video lectures, that is, learning by generating explanations in the presence of a co-learner will result in better learning performance even though the learning is not necessarily more demanding, and will result in more behaviors related to explanation adjustment and self-regulation.

## Data Availability

The data used to support the findings of this study are available from the corresponding author upon request.
